# Permission to pass: on the role of p53 as a gatekeeper for aneuploidy

**DOI:** 10.1007/s10577-023-09741-9

**Published:** 2023-10-21

**Authors:** Joana F. Marques, Geert J. P. L. Kops

**Affiliations:** 1 Royal Netherlands Academy of Arts and Sciences (KNAW), Hubrecht Institute, Uppsalalaan 8, 3584CT Utrecht, the Netherlands; 2 University Medical Center Utrecht, Heidelberglaan 100, 3584CX Utrecht, the Netherlands; 3 Oncode Institute, Jaarbeursplein 6, 3521AL Utrecht, the Netherlands

**Keywords:** Aneuploidy, p53, Cancer, Chromosomal instability

## Abstract

Aneuploidy—the karyotype state in which the number of chromosomes deviates from a multiple of the haploid chromosome set—is common in cancer, where it is thought to facilitate tumor initiation and progression. However, it is poorly tolerated in healthy cells: during development and tissue homeostasis, aneuploid cells are efficiently cleared from the population. It is still largely unknown how cancer cells become, and adapt to being, aneuploid. P53, the gatekeeper of the genome, has been proposed to guard against aneuploidy. Aneuploidy in cancer genomes strongly correlates with mutations in *TP53*, and p53 is thought to prevent the propagation of aneuploid cells. Whether p53 also participates in preventing the mistakes in cell division that lead to aneuploidy is still under debate. In this review, we summarize the current understanding of the role of p53 in protecting cells from aneuploidy, and we explore the consequences of functional p53 loss for the propagation of aneuploidy in cancer.

## Introduction

**Fig. 1:**
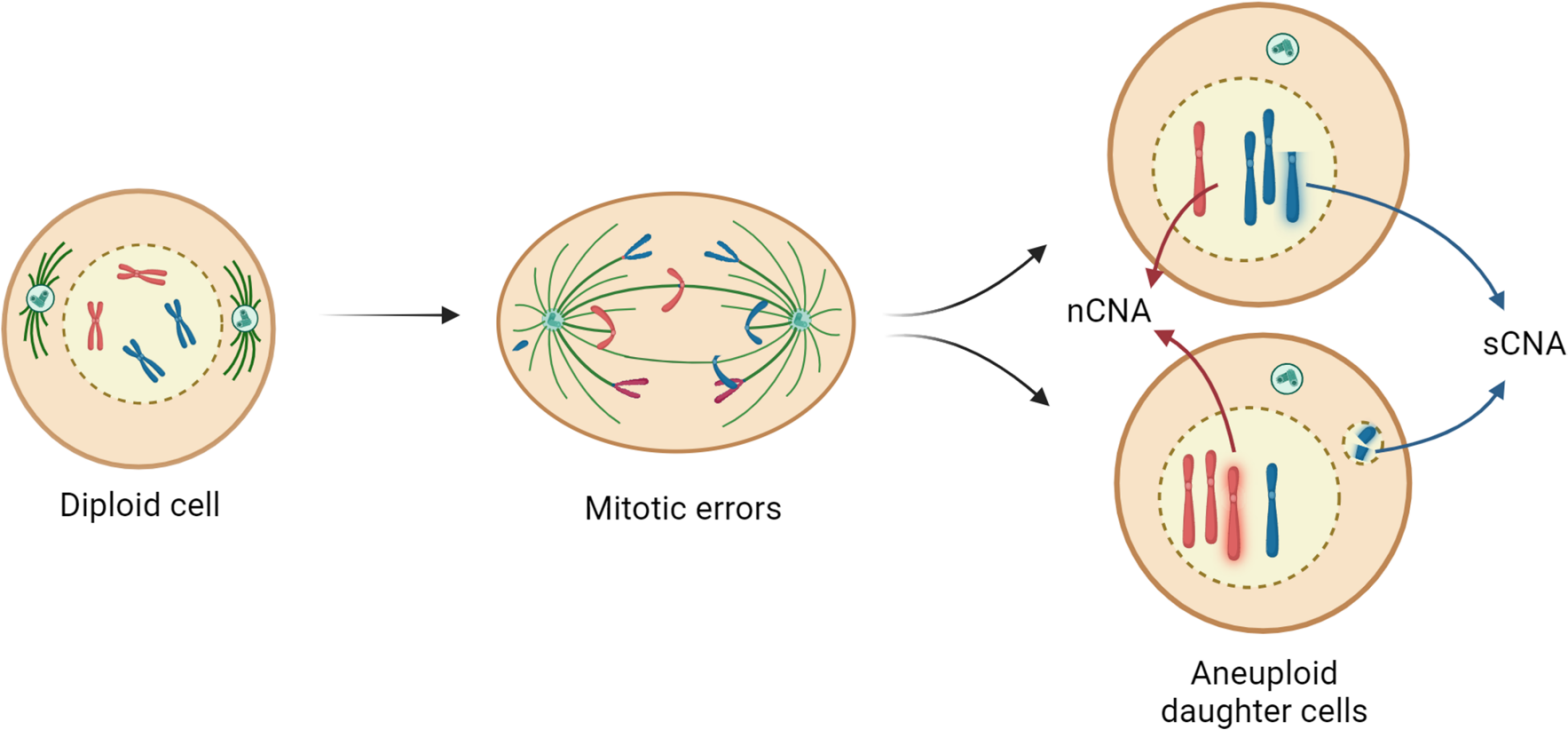
Aneuploidy: numerical vs. structural CNAs. Aneuploidy can arise from erroneous cell divisions. We define aneuploidy as a karyotype state in which the number of chromosomes deviates from a multiple of the haploid chromosome set. CNAs present in aneuploid cells can come in different flavors: numerical CNAs (nCNAs), which are whole-chromosome gains or losses, and structural CNAs (sCNAs), which are sub-chromosomal gains and losses ranging from arm-level alterations to smaller fractions of the genome (focal alterations)

Changes to the genome are rarely tolerated by healthy cells. They possess elaborate mechanisms to prevent accumulation of, for example, single nucleotide substitutions or copy number alterations (CNAs). CNAs can appear in various forms, most notably focal and arm-level CNAs (hereafter referred to as structural CNAs—sCNAs) and whole chromosome CNAs (hereafter referred to as numerical CNAs—nCNAs). sCNAs involve copy number changes to parts of chromosomes, while nCNAs involve gains or losses of entire chromosomes (Fig. 1). CNAs result mainly from errors in chromosome segregation during mitosis or meiosis, creating daughter cells with aneuploid karyotypes (Gordon et al. 2012; Sheltzer and Amon 2011). Aneuploidy—a karyotype state in which the genome deviates from a multiple of the haploid set of chromosomes—is highly prevalent in cancer (sCNA and/or nCNA are present in ~ 90% of tumor samples), where ~ 66% of all tumor samples from The Cancer Genome Atlas (TCGA) dataset carry at least one nCNA (Knouse et al. 2017; Taylor et al. 2018). Aneuploidy in cancer cells is a genomic manifestation of the increased rate at which cells mis-segregate chromosomes, a phenotype known as chromosomal instability (CIN). CIN is prevalent in cancer but its frequencies can vary greatly, even within the same tumor type (Lengauer et al. 1997; Bolhaqueiro et al. 2019). CIN not only leads to aneuploidy but also drives karyotype heterogeneity in tumor cell populations (Shoshani et al. 2021; Sansregret and Swanton 2017; Watkins et al. 2020; Bolhaqueiro et al. 2019). Depending on the degree of CIN, this can result in opposing effects on tumor initiation and progression: while CIN can accelerate tumor evolution by creating favorable karyotypes that increase cancer plasticity, fueling metastasis and therapy resistance (Gerstung et al. 2020; Sansregret and Swanton 2017; Watkins et al. 2020; Ben-David and Amon 2020; Chunduri and Storchová 2019), it can also suppress tumor initiation or progression in several mouse models when induced to high levels (Sheltzer et al. 2017; Godek et al. 2016; Rowald et al. 2016; Silk et al. 2013; Hoevenaar et al. 2020; Zasadil et al. 2016). Selection pressure for CNAs to gain or lose driver oncogenes or tumor suppressor genes, respectively, is one way in which the tumor-promoting effects are achieved (Davoli et al. 2013; Sack et al. 2018; Trakala et al. 2021; Shih et al. 2023). For example, in a lymphoma mouse model, CIN was followed by clonal selection of recurrent CNAs that confer a proliferative advantage (Shoshani et al. 2021; Trakala et al. 2021), including gains of any chromosome (natural or engineered) that carried the *c-Myc* gene (Trakala et al. 2021). As a result of selection for oncogenic drivers, whole chromosome CNA patterns are context-dependent, being conserved within tumors of a tissue type but divergent between tumors of different tissues (Sack et al. 2018; Hoadley et al. 2018; Ben-David and Amon 2020; Davoli et al. 2013).

Intriguingly, while aneuploidy is common in cancer, it is detrimental to cellular fitness (Sheltzer and Amon 2011; Ben-David and Amon 2020) and rare in healthy cells: less than 5% of cells across healthy tissues are aneuploid (Knouse et al. 2014; Chunduri and Storchová 2019). While much is known about how cancer cells circumvent the detrimental effects of mutations, much less is known about how they deal with aneuploidy. A prime candidate to guard against propagation of aneuploid cells is p53, a transcription factor with a central role in ensuring genome stability by responding to various stresses (Levine 2020). Inactivation of the *TP53* gene at early stages of tumorigenesis, irrespective of tumor type, emphasizes its broad role as a tumor suppressor (Gerstung et al. 2020). p53 is referred to as the guardian of the genome, and in line with this, strong co-occurrence of aneuploidy and *TP53* gene inactivation in cancer suggests that p53 plays a role in guarding against aneuploidy (Taylor et al. 2018; Ciriello et al. 2013). However, the interplay between aneuploidy and p53 in cancer is still poorly understood. In this review, we summarize the current understanding of the role of p53 activation in response to aneuploidy in healthy cells and explore the consequences of functional p53 loss for propagation of aneuploidy in cancer.

## *TP53* gene mutations and aneuploidy in cancer

Several large-scale cancer genome analyses showed that of all common oncogenic mutations, aneuploidy most strongly correlates with mutations in the *TP53* gene (Taylor et al. 2018; Donehower et al. 2019; Hoadley et al. 2014; Ciriello et al. 2013; Davoli et al. 2017; Zack et al. 2013). These analyses were performed on TCGA datasets, using a definition of aneuploidy that included not only numerical CNAs but also arm-level and/or focal CNAs. For most cancer types, occurrence of CNAs correlates with overall mutation burden, with *TP53* being the most significantly associated mutant gene (Taylor et al. 2018), and higher prevalence of *TP53* mutations correlates with higher frequencies of CNAs (Hoadley et al. 2014). When comparing, within cancer types, samples with mutations in *TP53* to those without, CNAs are enriched in the cancers carrying *TP53* mutations. This is irrespective of gains or losses (Donehower et al. 2019). Of note, since the definition of aneuploidy used in the analyses included sCNAs, it is unclear whether *TP53* mutations correlate with nCNAs. This is potentially relevant since events leading to sCNAs are expected to include DNA breaks and activate a p53 response, while those leading to nCNAs are not necessarily expected to do so. It would thus be of interest to re-assess *TP53* mutation status in relation strictly to nCNAs. Interestingly, evidence of whole-genome doubling (WGD)—the duplication of at least 50% of the current set of chromosomes—is seen in 30–40% of all cancers, and it is correlated with prior loss of p53 function in more than half of the cases (Gerstung et al. 2020; Bielski et al. 2018). For example, in TCGA datasets, a subgroup of colorectal cancer tumors was characterized by early loss of p53 and WGD (Kim et al. 2021). WGD is considered an unstable state that is often followed by accumulation of sCNAs and nCNAs (Gerstung et al. 2020). Overall, both *TP53* mutations and aneuploidy are prevalent in tumors and show intriguing correlations, which may signal causal links between the two.

## The role of p53 in maintaining genome stability

To better understand the correlation between *TP53* gene mutations and aneuploidy in cancer, we first look at p53’s role as the guardian of the genome. During interphase, p53 can respond to a plethora of cellular stresses, including DNA damage, oxidative stress, replication stress, and hypoxia (Gambino et al. 2013; Bieging et al. 2014). This response ensures that only cells with an intact genome progress into mitosis, which is widely regarded as a main reason for the tumor suppressor function of p53 (Liebl and Hofmann 2021; Boutelle and Attardi 2021; Janic et al. 2018). The various intricacies of the regulation and function of p53 have been extensively reviewed elsewhere (Kastenhuber and Lowe 2017; Kruiswijk et al. 2015); for the context of this review, we will briefly outline what is known about p53 activation and consequent outcomes in response to an insult.

**Fig. 2:**
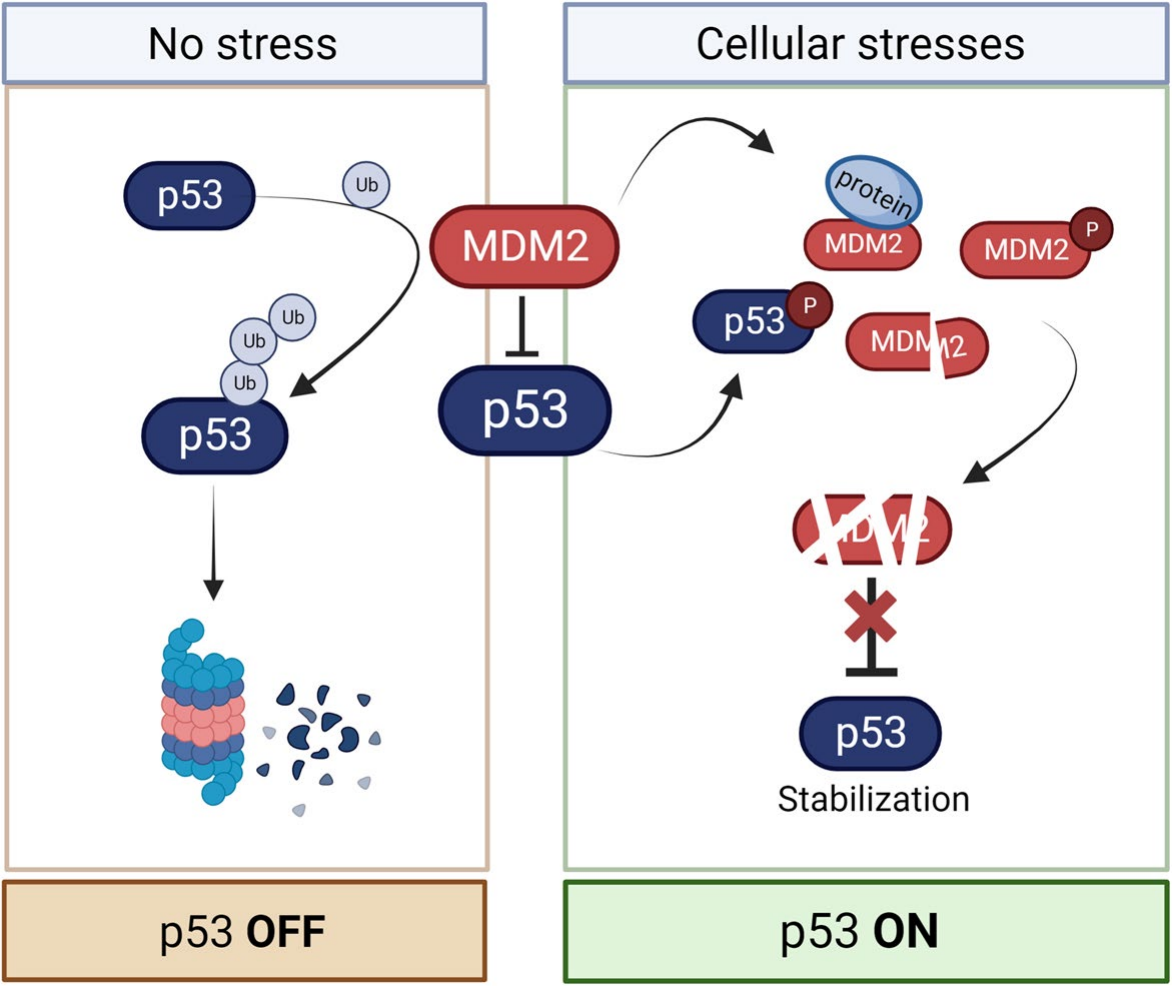
Regulation of p53. In physiological conditions, p53 interacts with its negative regulator MDM2, an E3 ubiquitin-protein ligase, which targets p53 for proteasomal degradation. Upon stress, upstream factors impinge on the p53-MDM2 interaction by inhibiting MDM2 function (e.g., target for degradation, phosphorylation of interaction site, cleavage, inhibition by interaction with other proteins (e.g., LATS1/2 or ribosome biogenesis subunits)). P53 can also be directly targeted, for example, by phosphorylation, to reduce affinity for MDM2. Disruption of MDM2-p53 interaction stabilizes p53 and leads to p53 activation in response to specific insults. Ub, ubiquitin.

In healthy cells, p53 is maintained at low levels by interaction with MDM2, which targets p53 for ubiquitin-dependent degradation (Fig. 2). Therefore, activation of p53, for example, in response to DNA damage, is achieved by disrupting this interaction, leading to p53 protein stabilization and regulation of target genes (Chène 2003; Levine 2020) (Fig. 2). DNA breaks activate the DNA damage response mediated by the ATM/CHK2 pathway, culminating in phosphorylation of p53 and MDM2 (Abuetabh et al. 2022; Banin et al. 1998; Chène 2003; Maya et al. 2001). Phosphorylation of p53 and MDM2 lowers the affinity for each other, resulting in p53 stabilization (Banin et al. 1998; Maya et al. 2001) (Fig. 2). The activation of the p53 pathway upon an insult can lead to distinct cell fates: cell cycle arrest, apoptosis, or senescence (Hafner et al. 2019). What determines a specific outcome upon p53 activation in response to, e.g., DNA damage depends on several variables. First, different tissues can express different isoforms of p53, expressed through alternative splicing. Although still poorly understood, different isoforms present distinct functions that in turn affect the outcome of p53 responses (Joruiz and Bourdon 2016). Second, post-translational modifications of the p53 protein, particularly phosphorylation, can significantly affect the degree of protein stability (via MDM2), conformation, localization, or affinity for binding partners (Liu et al. 2019). These modifications can also determine p53 transcriptional activity and consequently the cell fate: for example, while acetylation of p53 at residue K382 increases its activity after sustained DNA damage, a transient source of DNA damage leads to inhibition of p53 activity through methylation at the same residue (Loewer et al. 2010). Likewise, glucose deprivation can lead to G1 cell cycle arrest via p53: MDH1—an enzyme involved in glycolysis and mitochondrial respiration—stabilizes p53 by impinging on p53-MDM2 interaction and triggers post-translational modifications (acetylation and phosphorylation) that determine p53 activation (Lee et al. 2009; Kruiswijk et al. 2015; Wang et al. 2023). Third, p53 temporal dynamics can strongly determine cell fate (Jiménez et al. 2022; Purvis et al. 2012). Pulsatile p53 dynamics govern activation of p53 depending on the level of stress induced: while high levels of DNA damage lead to sustained p53 response and apoptosis, low levels of DNA damage result in a pulsed activation and milder effects, such as cell cycle arrest (Mönke et al. 2017; Purvis et al. 2012). Additionally, p53-dependent cell fate decisions hinge on pulsing activity of upstream and downstream players (Jiménez et al. 2022; Batchelor et al. 2008; Hanson et al. 2019; Paek et al. 2016; Stewart-Ornstein and Lahav 2017).

To summarize, the cellular stress response pathways commonly impinge on the regulation of the interaction between p53 and MDM2 (Fig. 2), and, although highly complex and not fully understood, their effect on p53 activation and cell fates depend on the level of stress and the context of the affected cell (Vousden and Prives 2009; Mönke et al. 2017).

## *TP53* mutations: a cause of aneuploidy?

**Fig. 3:**
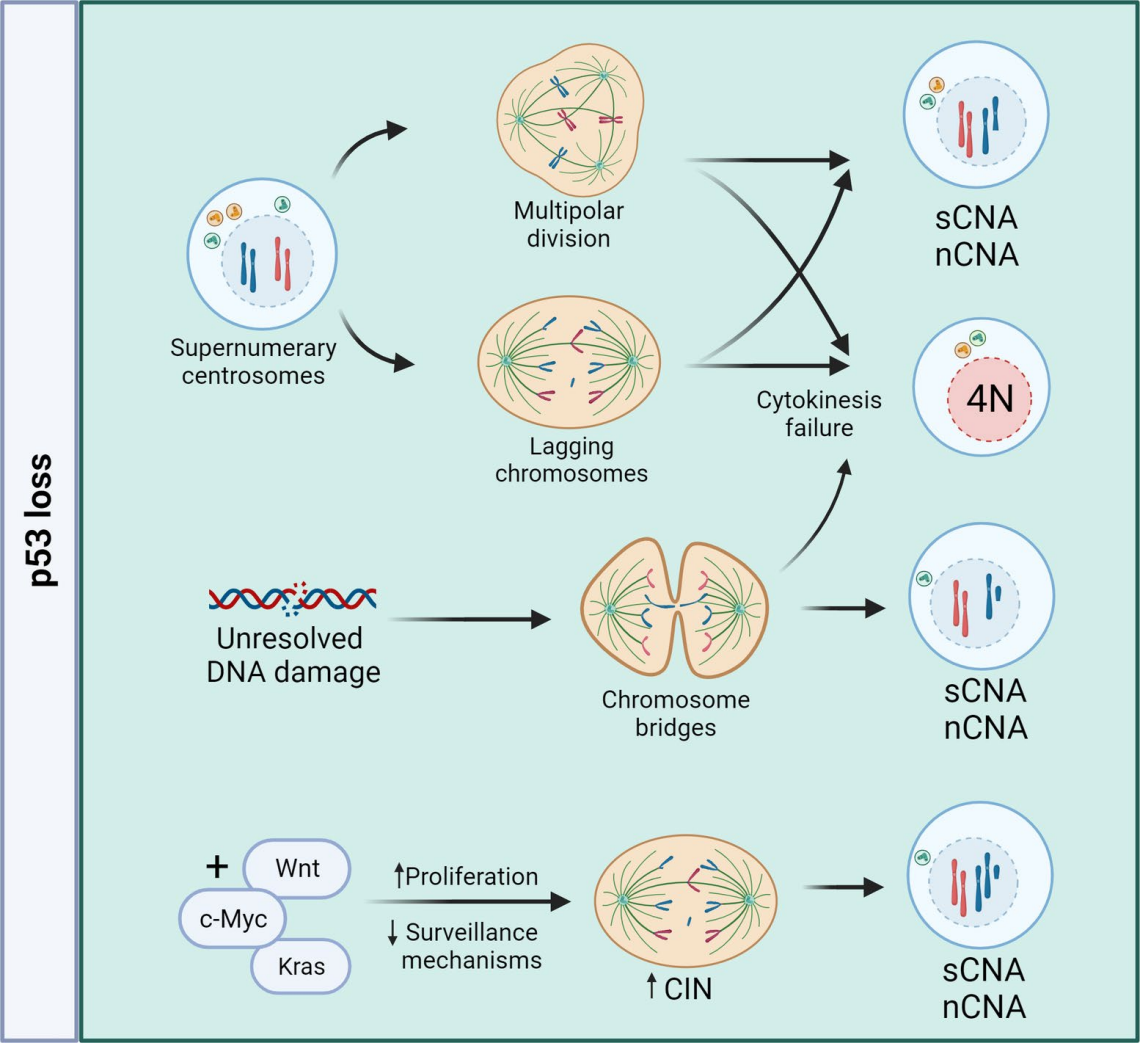
Consequences of p53 loss. Cells that lose p53 function can become aneuploid by various means, including supernumerary centrosomes, DNA damage, and altered proliferation and cellular surveillance mechanisms. Supernumerary centrosomes can result in multipolar spindles and/or lagging chromosomes, causing CNAs and WGD. Unresolved DNA damage can contribute to replication stress and mitotic errors (e.g., chromosome anaphase bridges) likely resulting in structural CNAs (sCNAs) or WGD. The combination of p53 loss with alterations in driver genes affects proliferation and surveillance mechanisms, resulting in aneuploid progeny with sCNA and/or numerical CNAs (nCNA)

The role of p53 in maintaining genome stability and its frequent inactivation in aneuploid cancers raises the possibility that loss of p53 can cause aneuploidy (Table 1; Fig. 3). In support of this, *TP53* knockout human intestinal organoids display CIN (Drost et al. 2015). This may be an indirect effect of the role of p53 in protecting cells from entering mitosis with damaged DNA (Fig. 3) (Bunz et al. 1998). Similarly, deletion of *TP53* in an acute myeloid leukemia (AML) cell line leads to CIN and aneuploidy (Cazzola et al. 2019). Recently, human gastric organoids carrying *TP53* gene mutations and followed for genome alterations over a 2-year period showed progressive and ordered accumulation of CNAs that are recurrently observed in gastric tumors, establishing a strong causal connection between *TP53* mutations and recurrent CNAs in cancer (Karlsson et al. 2023).

**Table 1:** P53 loss as a potential cause for aneuploidy

Model of study	Mode of p53 inactivation	CIN/aneuploidy	Mode of measuring CIN/aneuploidy	Other factors	Citations
Primary culture of mammary mouse model	*p53* + */* + ; *p53* + */ − *; *p53 − *(LOH); *p53 − / − *	No	Metaphase spreads	Combination with transgenic Wnt leads to aneuploidy	Donehower et al. (2019)
HCT116 (mostly); RKO; DLD1	*p53* shRNA and *p73* shRNA	No	IF to detect lagging chromosomes	Combination with loss of p73 caused CIN	Schmidt et al. (2021)
ACC-LC-176 near-diploid lung cancer and HCT116 (non-CIN control)	Human papilloma virus 16-E6-directed inactivation of p53	No	FISH	-	Haruki et al. (2001)
HCT116	*p53* + */* + *; p53 − / − *	No	FISH	Combination with loss of pRB promotes CIN and aneuploidy	Manning et al. (2014)
HCT116 and primary fibroblasts	*p53* KO by targeted homologous recombination	No (small tendency towards tetraploid)	M-FISH; FISH with centromeric probes	-	Bunz et al. (2002)
MEFs and lymphomas (in vivo)	*p53 − / − *or *p53* mutated at 515C (lack induction of apoptosis)	*p53 − / − :* Yes; *p53515C/515:* No	Flow cytometric (DNA content); metaphase spreads	Complete loss of p53 leads to centrosome amplification and sCNAs (mostly lymphomas)	Liu et al. (2004)
Human intestinal WT organoids	CRISPR-Cas9 mediated *p53* KO	CIN: Yes; Aneuploidy: No	CIN: live cell imaging; aneuploidy: metaphase spreads	Combination with loss of APC showed increase in aneuploidy	Drost et al. (2015)
184hTERT diploid breast epithelial cell line	CRISPR-Cas9 mediated *p53* KO	Yes (increased heterogeneity and fitness of aneuploid populations)	Whole genome single cell sequencing	-	Salehi et al. (2021)
MEFs	Germline *p53 − */* − *	Yes (cytokinesis failure and chromosomal abnormalities)	Live cell imaging; metaphase spreads; flow cytometry; FISH	Combination with loss of Rassf1a leads to stronger increase in ploidy (Loss of p53 alone leads mostly to lymphomas)	Tommasi et al. (2011)
Primary MEFs and spleen cells mouse intestinal tissue (in vivo)	Germline *p53 − / − *	Yes (lagging chromosomes)	Metaphase spreads in MEFs; IF for abnormal mitosis in intestinal tissue sections	Combination with *Cenpe* + */ − *	Funk et al. (2021)
Single cell suspensions of spleen, bone marrow, and thymus; spleen and skin fibroblasts (in vivo)	Germline *p53 − / − *	Yes (plus abnormal centrosome amplification)	Metaphase spreads	Combination with *c-Myc* overexpression	Fukasawa et al. (1997)
Pancreatic ductal adenocarcinoma (PDAC) mouse model	Germline *p53* + */ − *(with LOH)	Yes (mostly losses and WGD)	Single-cell genome sequencing; FISH	p53 loss is combined with *Kras*^*G12D*^ mutation	Baslan et al. (2022)
Lymphomas (in vivo)	Conditional *p53* knockout in T-cells (*p53* + */ − ; p53 − / −*)	Yes	aCGH, interphase FISH	Combination of p53 loss with Mps1 inhibition enhanced aneuploidy and tumor development	Foijer et al. (2014)
Lymphomas (in vivo)	Germline *p53 − / − *	Yes (nCNAs)	Metaphase spreads	Loss of p53 alone was enough to induce thymic lymphomas with nCNAs to similar levels of p53 loss combined with reduction of Bub1 protein levels	Baker et al. (2009)
Mouse mammary epithelial cells (MMECs); Xenografts models of tetraploid cells (in vivo)	Germline *p53 − / − *	Yes (diploid and tetraploid *p53 − / − *MMECs develop nCNA and sCNAs)	Flow cytometry (DNA content); metaphase spreads	WGD was induced by blocking cytokinesis (DCB treatment); diploid *p53 − / − *cells become spontaneously tetraploid in culture	Fujiwara et al. (2005)
Biopsies of patients with BE; BAR-T and BAR-T10 BE patient-derived cell lines	*p53* mutations (dysplastic samples); *p53* siRNA	Yes (plus abnormal centrosome number)	Centrosome/centriole number and DNA content analysis by IF	-	Lopes et al. (2018)

Summary of literature that examined the development of aneuploidy upon p53 loss. The table includes the strategies used to induce p53 inactivation, whether aneuploidy was detected when only p53 was lost and methods used to measure aneuploidy. Where relevant, co-occurrence of other factors that contributed to aneuploidy development is noted. *LOH*, loss of heterozygosity; *IF*, immunofluorescence; *FISH*, fluorescence in situ hybridization; *M-FISH*, multiplex-FISH; *CIN*, chromosomal instability; *MEFs*, mouse embryonic fibroblasts; *WGD*, whole genome doubling; *DCB*, dihydrocytochalasin-B; *aCGH*, array comparative genomic hybridization; *shRNA*, short hairpin RNA; *KO*, knockout; *BE*, Barrett’s esophagus; *WT*, wild type

How might loss of p53 cause aneuploidy? Mouse embryonic fibroblasts lacking p53 have amplified centrosomes, as a result of misregulated CDK2/cyclin-E compromising the centrosome duplication cycle (Fukasawa et al. 1996; Liu et al. 2004; Tarapore et al. 2001). Interestingly, likely due to a more stringent regulation of cyclin-E, loss of p53 in human non-transformed cells does not lead to centrosome amplification (Kawamura et al. 2004). Yet there is an intriguing correlation between p53 loss and supernumerary centrosomes in human cancers (Lopes et al. 2018; Fukasawa 2005; Chan 2011). Supernumerary centrosomes can lead to CIN by creating multipolar spindles that predispose cells to lagging anaphase chromosomes, resulting in aneuploid progeny (Silkworth et al. 2009; Ganem et al. 2009) (Table 1; Fig. 3). In agreement with this, cells in human *TP53* knockout hepatocyte organoids display multipolar spindles (Artegiani et al. 2020). Loss of p53 can also predispose cells to WGD by allowing the survival of cells that underwent cytokinesis failure (Baslan et al. 2022; Fujiwara et al. 2005; Tommasi et al. 2011). An additional mechanism may involve cytokinesis failure as a result of DNA damage-induced anaphase bridges at the cleavage furrow (Andreassen et al. 2001a, b; Bunz et al. 1998; Bakhoum et al. 2017; Janssen et al. 2011) (Table 1; Fig. 3). WGD can further promote CIN: in most cancers with evidence of early WGD, subsequent near-triploidy and extensive CNAs are common (Gerstung et al. 2020; Knouse et al. 2017; Storchova and Pellman 2004; Storchova and Kuffer 2008; Baslan et al. 2022; Dewhurst et al. 2014; Zeng et al. 2023).

Contrasting these observations are studies reporting that *TP53* mutations are not sufficient to cause aneuploidy: monolayer cancer or non-transformed cell lines often remain diploid after p53 deletion/inactivation (Simoes-Sousa et al. 2018; Bunz et al. 2002; Haruki et al. 2001). Loss of p53 in HCT116 cells induces CIN only when combined with the loss of p73 (Schmidt et al. 2021) or loss of pRB (Manning et al. 2014), and the CIN seen upon *TP53* knockout in human intestinal organoids was not accompanied by a significant increase in aneuploidy (Drost et al. 2015) (Table 1).

What could explain the seemingly contradictory findings about the appearance of aneuploid cells upon p53 inactivation? *TP53* knockout in an AML cell line resulted in appearance of aneuploid cells containing both sCNAs and nCNAs (Cazzola et al. 2019). Conversely, cells with functional p53 can accumulate some nCNAs but rarely sCNAs. In line with this, inducing CIN in p53-proficient HCT116 or RPE1 cells resulted in cycling aneuploid cells with mostly whole-chromosome gains (e.g., trisomies), while most whole-chromosome losses and sCNAs could be detected only when p53 was inactivated (Soto et al. 2017; Chunduri et al. 2021). So, like the analysis of cancer genome datasets, the discrepancy may lie in the definition of aneuploidy: even though nCNAs survive better in p53-inactivated cells, it is possible that loss of p53 leads predominantly to sCNAs rather than nCNAs.

In vivo p53-deficient germline mouse models display CIN and aneuploidy in tissues such as lymphoid (thymus, bone marrow, and spleen) (Fukasawa et al. 1997; Funk et al. 2021) and intestine (Funk et al. 2021) (Table 1). Likewise, inducible loss of p53 in mouse esophagus progenitor cells leads to accumulation of giant poly-aneuploid like cells—aneuploid cells that have undergone WGD (Murai et al. 2022). Nevertheless, oncogenic transformation in vivo upon p53 loss happens predominantly in blood cells (lymphomas) and/or connective tissue (sarcomas), and these cancers are aneuploid (Funk et al. 2021; Liu et al. 2004; Tommasi et al. 2011; Chi et al. 2009; Baker et al. 2009; Foijer et al. 2014) (Table 1). Induction of CIN by deleting the spindle assembly checkpoint (SAC) kinases Mps1 or Bub1 in mice heterozygous for functional p53 (*p53* + */ −*) accelerated loss of heterozygosity (*p53 − / −*) and the development of aneuploid lymphomas (Foijer et al. 2014; Baker et al. 2009) (Table 1). Other in vivo p53 mutant cancer models also show aneuploidy, but loss of p53 was often combined with additional oncogenic events. Examples include transgenic *Wnt* overexpression (mammary cancer) (Donehower et al. 1995), *Kras* mutation (pancreatic cancer) (Baslan et al. 2022), or spontaneous *c-Myc* overexpression (lymphoma) (Fukasawa et al. 1997) (Table 1, Fig. 3). The combination of multiple factors makes it challenging to dissect the contribution of loss of p53 alone to the development of aneuploidy in these tissues.

To summarize, depending on the model and conditions (species, culture or tissue type, additional oncogenic alterations), p53 loss by itself can cause aneuploidy. Whether this is a result of increased CIN or of a higher probability of survival of aneuploid cells in vivo is unclear. Resolving this requires single cell analysis of CIN and aneuploidy in vitro and in vivo upon acute inactivation of p53.

## A healthy cell’s response to aneuploidy

*TP53* mutations correlate with aneuploidy in cancer, but as outlined earlier, it is unclear whether p53 loss can directly cause chromosome segregation errors. Moreover, loss of functional p53 does not invariably lead to aneuploidy. Instead, loss of p53 might be a permissive characteristic for propagation of aneuploidy. In order to explore this possibility we will first outline how healthy cells respond to aneuploidy.

### Aneuploidy-induced stresses

Aneuploidy is rare in healthy cells (Knouse et al. 2014; Chunduri and Storchová 2019). Cells are protected from becoming aneuploid by various cell cycle checkpoints (e.g., replication or chromosome segregation checkpoints) (Chunduri and Storchová 2019; Gaillard et al. 2015; McAinsh and Kops 2023), and if somehow cells escape such checkpoints and become aneuploid, they appear to be eliminated from the population. Immune surveillance can drive the elimination of senescent aneuploid cells, mostly mediated by natural killer cells (Santaguida et al. 2017; Wang et al. 2021). Whether immune cells can recognize specifically aneuploid cells, and if so how, is largely unknown. Aneuploidy induces severe stresses that cause a substantial fitness decline (Sheltzer et al. 2017; Wang et al. 2021; Santaguida et al. 2017; Zhu et al. 2018; Gordon et al. 2012; Sheltzer and Amon 2011; Torres et al. 2007; Williams et al. 2008). As extensively reviewed elsewhere (Chunduri and Storchová 2019; Gordon et al. 2012; Zhu et al. 2018), aneuploidy can cause DNA damage (Janssen et al. 2011; Sheltzer et al. 2011; Passerini et al. 2016), replication stress (Ohashi et al. 2015; Passerini et al. 2016; Garribba et al. 2023), proteotoxic stress (Ohashi et al. 2015; Santaguida and Amon 2015b; Oromendia et al. 2012; Torres et al. 2007), hypo-osmotic stress (Tsai et al. 2019), metabolic stress (Stingele et al. 2012; Foijer et al. 2014), lysosomal stress (Santaguida and Amon 2015a; Santaguida et al. 2015), and an inflammatory response (Santaguida et al. 2017; Wang et al. 2021). These often lead to cell cycle arrest and/or cell death. The mechanisms by which aneuploidy causes these stresses are not completely resolved, but have been elucidated in some cases. Aneuploid cells can accumulate DNA damage as a result of DNA breaks on lagging chromosomes or anaphase bridges, or from mis-segregated chromosomes that end up in micronuclei, which are prone to rupture and exposure of the entrapped chromosome to cytoplasmic exonucleases (Umbreit et al. 2020; Zhang et al. 2015; Janssen et al. 2011; Hatch et al. 2013). Through imbalances in gene content from nCNAs, aneuploidy can affect transcription and translation (Chunduri and Storchová 2019), leading to, for example, a shortage of replication factors (such as MCM2–7) that in turn induce replication stress and cause DNA damage in under-replicated regions (Passerini et al. 2016; Garribba et al. 2023). Although dosage compensation mechanisms exist in aneuploid cells to negate the protein imbalances that result from chromosome gains (Stingele et al. 2012), they suffer from proteotoxic stress: imbalances in protein complex stoichiometries put a strain on protein folding and degradation machineries, leading to aggregation of misfolded proteins in the cytoplasm (Chunduri and Storchová 2019). Interestingly, aneuploidy-induced stresses are not the same for all types of CNAs, given that, for example, monosomies do not induce proteotoxic stress (Chunduri et al. 2021). Therefore, further efforts are needed to elucidate the mechanisms by which CNAs trigger the aforementioned stress responses.

### p53 pathway response to aneuploidy

The overlap in stresses that arise from aneuploidy and those that activate p53 raises the question whether p53 is activated in response to aneuploidy and hence may impact the fate of aneuploid cells. Indeed, several studies have shown that p53 can be activated in response to CIN and/or aneuploidy (Table 2). This can occur regardless of the strategy used to induce CIN. For example, disrupting the SAC, alone or in combination with disrupting chromosome congression (by inhibiting MPS1), the master kinase of the SAC, − / + the mitotic kinesin CENP-E) in RPE-1 or HCT116 cells leads to sCNAs and nCNAs followed by p53 activation and consequent cell cycle arrest (Santaguida et al. 2017; Soto et al. 2017; Narkar et al. 2021; Janssen et al. 2011; Simoes-Sousa et al. 2018; Li et al. 2010) (Table 2). Similarly, induction of WGD in RPE-1 cells also causes cell cycle arrest mediated by p53 (Crockford et al. 2017; Ganem et al. 2014; Gemble et al. 2022), and mitotic arrest-and-release strategies induce p53 activation after mis-segregation events and result in cell cycle arrest (Thompson and Compton 2010; Dalton et al. 2010). P53 activation has also been observed in cell lines derived from mouse models of CIN (Li et al. 2010; Silk et al. 2013). However, aneuploidy does not invariably lead to p53 activation in monolayer cultures. For example, certain nCNAs or low levels of aneuploidy can propagate in p53-proficient RPE-1 cells (Soto et al. 2017; Santaguida et al. 2017), and even high aneuploidy levels can lead to cell cycle arrest in a p53-independent manner (Santaguida et al. 2017).

**Table 2:** p53 responses to aneuploidy

Cell type/mouse genotype	Mode of CIN/aneuploidy induction	CIN/aneuploidy phenotype	DNA damage	p53 activation	Mode of measuring p53 activation	Citations
EEB (transformed acute myeloid leukemia cell line)	Spontaneous; MPS1i^a^ + CENP-Ei^b^	p53 WT cells: nCNAs	Yes	No (only tested in cycling aneuploid cells with nCNAs)	Western blot	Cazzola et al. (2019)
*2D:* RPE-1, HCT116; Nalm6 (suspension cell line)	MPS1i^a^; nocodazole	MPS1i: nCNAs; nocodazole: multipolar divisions	No	Yes	Western blot; IF	Narkar et al. (2021)
*3D:* hMO and mCO				No	Western blot	
RPE-1 cells	MPS1i^a^,^c^ + CENP-Ei^b^	sCNAs and nCNAs; micronucleus formation	NA	Yes (in ~ 16% of cells)	IF	Soto et al. (2017)
RPE-1 cells	MPS1i^a,d^	Arrested cells: genomic imbalances involving more than 20% of their genomes (sCNA in 47% of cells and also nCNAs)	Yes	Yes (50% of arrested cells)	Western blot; IF	Santaguida et al. (2017)
		Cycling cells: genomic imbalances involving less than 5% of their genomes (sCNA in 18% of cells and also nCNAs)	Inconclusive	No		
HCT116	MPS1i^e^; MPS1i^e^ + CENP-Ei^b^ to minimize DNA damage	Mostly nCNAs; 4N	No	Yes	Western blot; time-lapse imaging of GFP-p53 fusion biosensor	Simoes-Sousa et al. (2018)
RPE-1 cells	*Cytokinesis failure:* siRNA mediated KD of anillin; *Chemical inhibition:* Aurora Bi^f^; mitotic arrest with colcemid	WGD	No (only in micronuclei)	Yes	Real-time qPCR and Western blot	Potapova et al. (2016)
RPE-1 cells	MPS1i^d^	All cells became aneuploid (mostly nCNAs and a small fraction of sCNAs)	Yes	Yes	RNA sequencing	(Garribba et al. 2023)
RPE-1 cells	DCB; siRNA-mediated depletion of ECT2; Aurora Bi^g^	WGD	No	Yes	IF and Western blot	Ganem et al. (2014)
HCT116 and IMR90	Mitotic arrest (Nocodazole)	Mitotic slippage and increase in ploidy	Yes	Yes	IF	Dalton et al. (2010)
HCT116	Monastrol washout strategy; MAPK siRNA	Monastrol washout strategy—33% whole chromosomes; MAPK siRNA—33% lagging chromosomes	No	Yes	IF and Western blot	Thompson and Compton (2010)
HCT116	MPS1i^d^ and siRNA screening	Chromosome gains	-	Yes	Western blot	Lopez-Garcia et al. (2017)
MEFS derived from Cdc20^+/AAA^ mice; HCT116	*MEFs:* Cdc20 with deficient binding for Mad2 (impaired SAC); *HCT116:* siRNA depletion of MAD2, BUBR1, and CENP-E	FISH for p53 high cells: aneuploidy 5% for chromosome 4 and 10% for chromosome 10	No	Yes (mediated by ROS)	IF and Western blot	Li et al. (2010)
RPE-1; U2OS; MCF7; SW480 and BJs	Monastrol washout strategy; MPS1i^c^	Mostly lagging chromosomes	Yes	Yes	Western blot	Janssen et al. (2011)
RPE-1, BJ and, HCT116	Cyclin B1, actin or cyclin A2 depletion	WGD	Yes	Yes	Western blot	Gemble et al. (2022)
RPE-1 cells overexpressing cyclin D1; spontaneous 4N HCT116 cells	DCB	WGD	No (absence of p53 phosphorylated in Ser15)	Yes	Western blot	Crockford et al. (2017)

Summary of literature investigating p53 activation upon induction of aneuploidy along with the methods used to induce aneuploidy and to measure p53 activation. Detection of DNA damage is also included. *mCO*, mouse colon organoids; *hMO*, human mammary organoids; *MEFs*, mouse embryonic fibroblasts; *ROS*, reactive oxygen species; *DCB*, dihydrocytochalasin-B; *IF*, immunofluorescence; *siRNA*, small interfering RNA; *WT*, wild type

^a^MPS1 inhibitor NMS-P715

^b^CENP-E inhibitor GSK923295

^c^MPS1 inhibitor Cpd-5

^d^MPS1 inhibitor Reversine

^e^MPS1 inhibitor AZ3146

^f^Aurora B inhibitor ZM447439

^g^Aurora B inhibitor hesperadin

Additionally, a recent study using 3D cultures further challenged the hypothesis that p53 is activated in response to aneuploidy (Table 2). While p53 activation and cell cycle arrest upon chromosome mis-segregation was seen in monolayer cell cultures, no p53 activation was seen in either mouse colon or human mammary 3D organoid cultures (Narkar et al. 2021). In this study, chromosome segregation errors in the organoids were not accompanied by DNA damage, which may in part explain the lack of a p53 response and is in line with the requirement for p53 inactivation in the accumulation of sCNAs, as described earlier. Another possibility is that, since tissue architecture was proposed to be essential for chromosome segregation fidelity (Knouse et al. 2018), this may also somehow impact the p53 response. However, growing HCT116 cells in 3D did not alter the p53 response (Narkar et al. 2021), although it is debatable to what extent such cancer cells grown in 3D recapitulate tissue architecture. A point of consideration is the components of the medium used for organoid culture that might affect cellular responses: in human organoid lines specifically, p38 inhibitors are often used which might inhibit aneuploidy-induced stress response mediated by p38–p53 activation (Simoes-Sousa et al. 2018). Nonetheless, the 3D culture study emphasizes the importance of examining whether p53 is activated in response to aneuploidy in vivo. To our knowledge, while p53 activation has been detected in fibroblasts derived from mouse cancer models of CIN/aneuploidy, it has not been examined directly in vivo or in tissues of such models. Interestingly, p53 activation and elimination of aneuploid cells in mouse brains upon induction of CIN were reported in the context of mouse embryonic development (Shi et al. 2019).

Taken together, the inconsistent observations of a p53 response to aneuploidy in monolayer cultures and the lack of p53 response observed in 3D organoid cultures expose a gap in our understanding of the mechanisms by which cells respond to and surpass aneuploidy-induced stresses and the role of p53 in this.

## The mechanisms of p53 activation by aneuploidy

The observation that aneuploidy, at least in monolayer cultures, often results in p53 activation raises the question of the underlying mechanism. We envision two possibilities: either p53 senses aneuploidy per se or it senses the stresses that result from aneuploidy.

### Direct sensing of aneuploidy by p53

The first possibility is supported by a study reporting that cells sense lagging or misaligned chromosomes by marking them with phosphorylation of histone 3 (H3.3) on serine at position 31 (Ser31), which in turn activates p53 by an unknown mechanism (Hinchcliffe et al. 2016). Ser31 of H3.3 can be phosphorylated by the DNA damage response kinase CHK-1 in cancer cells that rely on the alternative lengthening of telomeres (ALT) pathway (Chang et al. 2015), although in the aforementioned study p53 activation from H3.3. Ser31 phosphorylation was apparently independent of DNA damage (Hinchcliffe et al. 2016). Alternatively, H3.3 Ser31 can be phosphorylated by the mitotic kinase Aurora B (Li et al. 2017), which is active at the cell’s midzone during anaphase and telophase (Fuller et al. 2008) and as such might mark a lagging chromosome. Paradoxically, however, one study showed that Aurora B can phosphorylate p53 at centromeres, resulting in accelerated p53 degradation (Gully et al. 2012). It thus remains unclear how H3.3-Ser31 is phosphorylated on mis-segregated chromosomes or how that impacts p53, and no subsequent studies have addressed this. P53 has also been reported to respond directly to mitotic defects that are connected to prolonged mitosis: a pool of p53 (phosphorylated at Ser15) located at centrosomes is released in the cytoplasm upon centrosome fragmentation during mitosis and recruits 53BP1, that in turn activates a mitotic surveillance pathway composed of 53BP1 and USP28 (Contadini et al. 2019). Similarly, a study proposed the existence of a “stopwatch” composed of USP28, 53BP1, and p53 that limits the proliferation of daughter cells that arise after a prolonged mitosis (Meitinger et al. 2016). A new pre-print study suggests that this “stopwatch” is a result of gradual MDM2 degradation that eventually leads to p53 activation in the following G1 (Fulcher et al. 2023). p53 has also been proposed to participate in a “tetraploidy checkpoint” in G1, able to detect and limit the proliferation of tetraploid cells. This concept resulted from the observation that newly formed tetraploid cells (generated by chemical induction of cytokinesis failure) would arrest in G1 in a p53-dependent manner (Andreassen et al. 2001a, b). However, the existence of such a checkpoint was challenged by other studies showing that G1 cell cycle arrest was not an obligatory outcome in tetraploid cells (Uetake and Sluder 2004; Wong and Stearns 2005). Taken together, the evidence for p53 as a direct sensor of the aneuploid state is currently thin.

### Indirect sensing of aneuploidy by p53

**Fig. 4:**
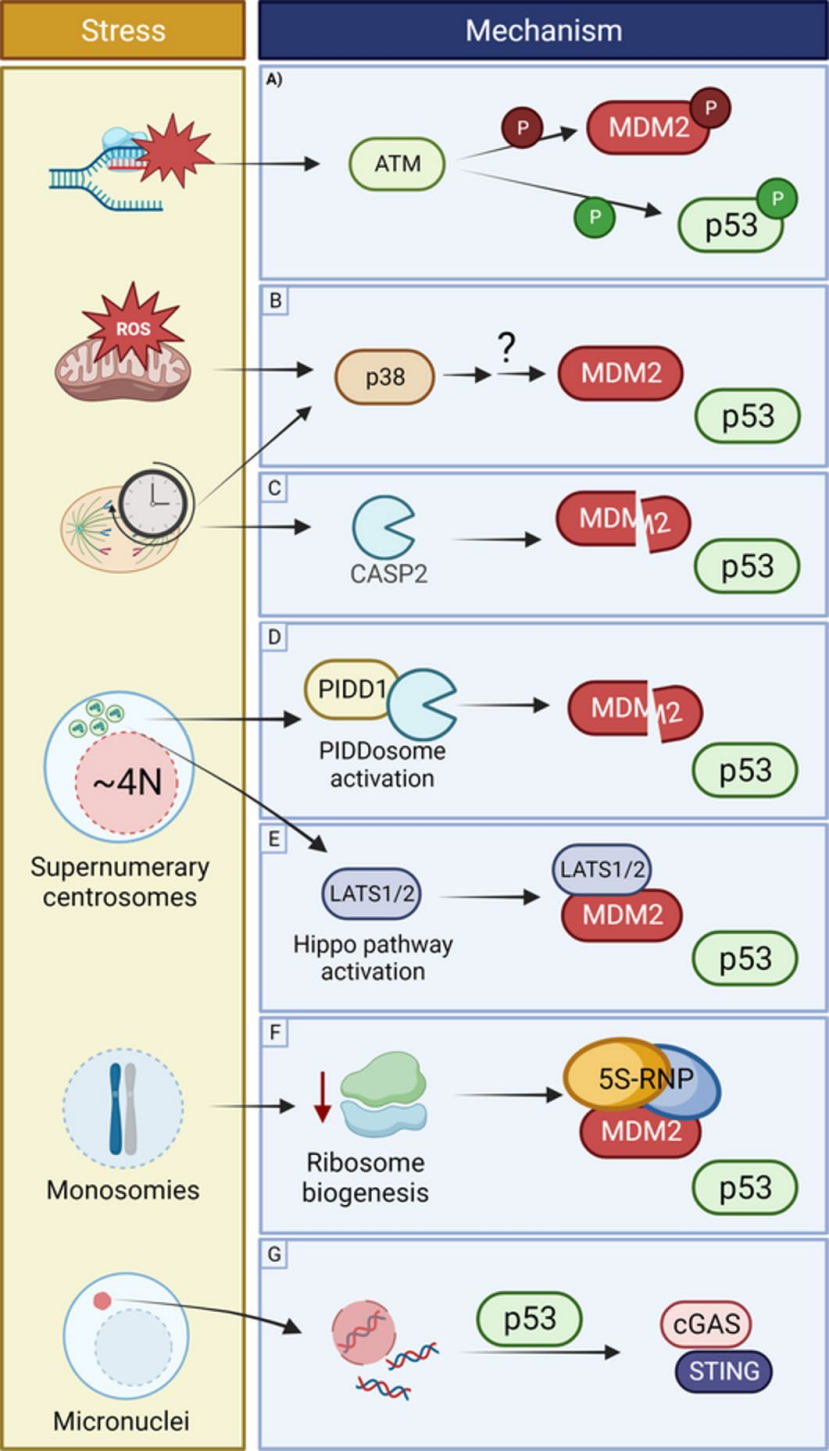
P53 activation in response to aneuploidy-induced stresses. Aneuploidy-induced stress responses often impinge on p53 activation by disrupting p53-MDM2 interaction. **A** Replication stress and DNA damage activate ATM, which can directly phosphorylate MDM2 or p53. **B** Metabolic stress and prolonged mitosis can trigger a p38 stress response, which indirectly targets MDM2 for degradation. **C** Prolonged mitosis can also activate CASP2, which can cleave MDM2. (**D**) Supernumerary centrosomes, often co-occurring with WGD, can trigger the PIDDosome (mediated by CASP2) or (**E**) the Hippo pathway: LATS1/2 binds MDM2 and reduces its affinity for p53. (**F**) Monosomies cause deficient ribosome biogenesis, which creates imbalances in ribosomal proteins, some of which then interact with MDM2 and reduce its affinity for p53. Although poorly understood, (**G**) the presence of cytoplasmatic DNA triggers p53 activation which in turn mediates the activation of the cytoplasmatic DNA sensing machinery (cGAS/STING) for immune activation

In the second possibility, p53 senses the stresses that result from aneuploidy (Fig. 4). The observation that low levels of aneuploidy do not activate p53 but higher aneuploidy levels do lead to p53 activation, cell cycle arrest, and senescence supports this notion (Li et al. 2010; Santaguida et al. 2017). Although there is currently little evidence for it, cells with low CIN levels in vivo may escape p53 surveillance mechanisms, while those with high CIN levels may elicit a p53-mediated apoptosis response, with differential outcomes on tumorigenesis (Li et al. 2010). If indeed p53 is activated in response to aneuploidy-induced stresses, there is likely a threshold for p53 activation upon a mis-segregation event (Santaguida et al. 2017; Soto et al. 2017), much like the thresholds proposed to dictate p53 response dynamics and cell fates upon DNA damage (Mönke et al. 2017; Loewer et al. 2010; Paek et al. 2016). A “just right” level of aneuploidy might then be sufficiently high to promote tumorigenesis while being sufficiently low to allow escape from p53-mediated cell cycle arrest or apoptosis.

As said, there is substantial overlap between stresses known to activate p53 and those elicited by aneuploidy. Are all of them part of the mechanism by which aneuploidy triggers p53 activation, or do some contribute more than others? This question is largely unanswered. The clearest link between aneuploidy-induced stress and p53 activation is again DNA damage, as described earlier (Fig. 4). CIN and WGD commonly cause DNA damage with ensuing p53 activation (Janssen et al. 2011; Santaguida et al. 2017; Soto et al. 2017; Dalton et al. 2010; Krzywicka-Racka and Sluder 2011; Kuffer et al. 2013; Ganem et al. 2014; Gemble et al. 2022). The fact that DNA damage underlies sCNAs (Janssen et al. 2011; Li et al. 2010; Santaguida et al. 2017) and that sCNAs are eliminated in p53-proficient cells (Soto et al. 2017; Dalton et al. 2010; Cazzola et al. 2019) suggests that aneuploidy-associated DNA damage, at least in part, explains the p53 response to aneuploidy. In fact, loss of p53 or DNA damage repair genes (e.g., *MLH1* and *MSH2*) lead to accumulation of sCNAs and single nucleotide alterations, suggesting that p53 acts mostly through the DNA damage response to prevent propagation of sCNAs (Janic et al. 2018). Although likely, for most of the other aneuploidy-related stresses like metabolic stress, replication stress, hypo-osmotic stress, proteotoxic stress, and autophagy stress, there is currently no evidence that they are a crucial intermediate between aneuploidy and p53. Examining this requires assessment of p53 activity (dynamics) following aneuploidy after specific elimination of one type of stress. This would also help understanding how p53 limits the survival of cells that acquire nCNAs, since nCNAs, in contrast to sCNAs, are less likely to accumulate DNA breaks. Of note, for one type of nCNA the mode of p53 activation seems resolved: monosomies are incompatible with functional p53. This is likely due to defects in ribosome biogenesis (Chunduri et al. 2021). Because ribosomal genes are spread across the genome, monosomies cause imbalances in ribosomal protein complexes, some of which can interact with MDM2 and activate the p53 response (Lindström et al. 2022) (Fig. 4). Some DNA damage-independent mechanisms have been proposed to link CNAs to p53 activation (Fig. 4). p38 works side by side with p53 to limit progression of cells in response to stress stimuli. Upon mis-segregation events, p38 causes apoptosis, at least in part through p53 stabilization and suppression of HIF-1α (a master regulator of the hypoxia response) (Simoes-Sousa et al. 2018). P38 has been shown to indirectly target MDM2 (unknown mechanism) leading to p53 stabilization (Zhang et al. 2021) (Fig. 4). Similarly, a postmitotic stress response by p38–p53 can trigger p53-dependent cell cycle arrest in G1 after prolonged mitosis or mitotic slippage without proper cytokinesis (Uetake and Sluder 2018; Vogel et al. 2004). In *Drosophila*, the p38–p53 axis participates in the response to metabolic stress induced by ROS formation as a consequence of CIN (Clemente-Ruiz et al. 2016). Recently, p53 was linked to the activation of the cytosolic DNA-sensing cGAS/STING pathway (Ghosh et al. 2023). Micronuclei formed after a mis-segregation event are prone to rupture, leading to accumulation of cytosolic DNA that can trigger cGAS/STING activation (Kwon et al. 2020) (Fig. 4). In the presence of cytosolic DNA, p53 induces the degradation of the exonuclease TREX1 (a DNA degrading enzyme), leading to the accumulation of cytosolic DNA and consequent detection by the sGAS/STING pathway (Ghosh et al. 2023). P53 can also limit proliferation of cells with supernumerary centrosomes after WGD (Ganem et al. 2009; Darp et al. 2022) through activation of the Hippo pathway (Ganem et al. 2014) or the PIDDosome (a caspase-2 activator) (Ganem et al. 2009; Fava et al. 2017) (Fig. 4). Activation of CASP2 (caspase-2) in response to CNAs, mitotic delay or DNA damage during mitosis, causes MDM2 cleavage, p53 stabilization, and thus aneuploid cell clearance through mitotic cell death (Fig. 4) (Dawar et al. 2017; Lopez-Garcia et al. 2017; Castedo et al. 2004; Lim et al. 2021). In colorectal cancer cell lines, repression of CASP2 activity by loss of BCL9L (a component of Wnt signaling pathway) allowed survival of CIN cells (Lopez-Garcia et al. 2017). In summary, several mechanisms can lead to p53 activation upon a mis-segregation event in vitro. However, more studies are necessary to clarify both the mechanisms of activation of p53 and the outcomes thereof in response to aneuploidy in 3D and in vivo.

## Conclusions and future outlook

**Fig. 5:**
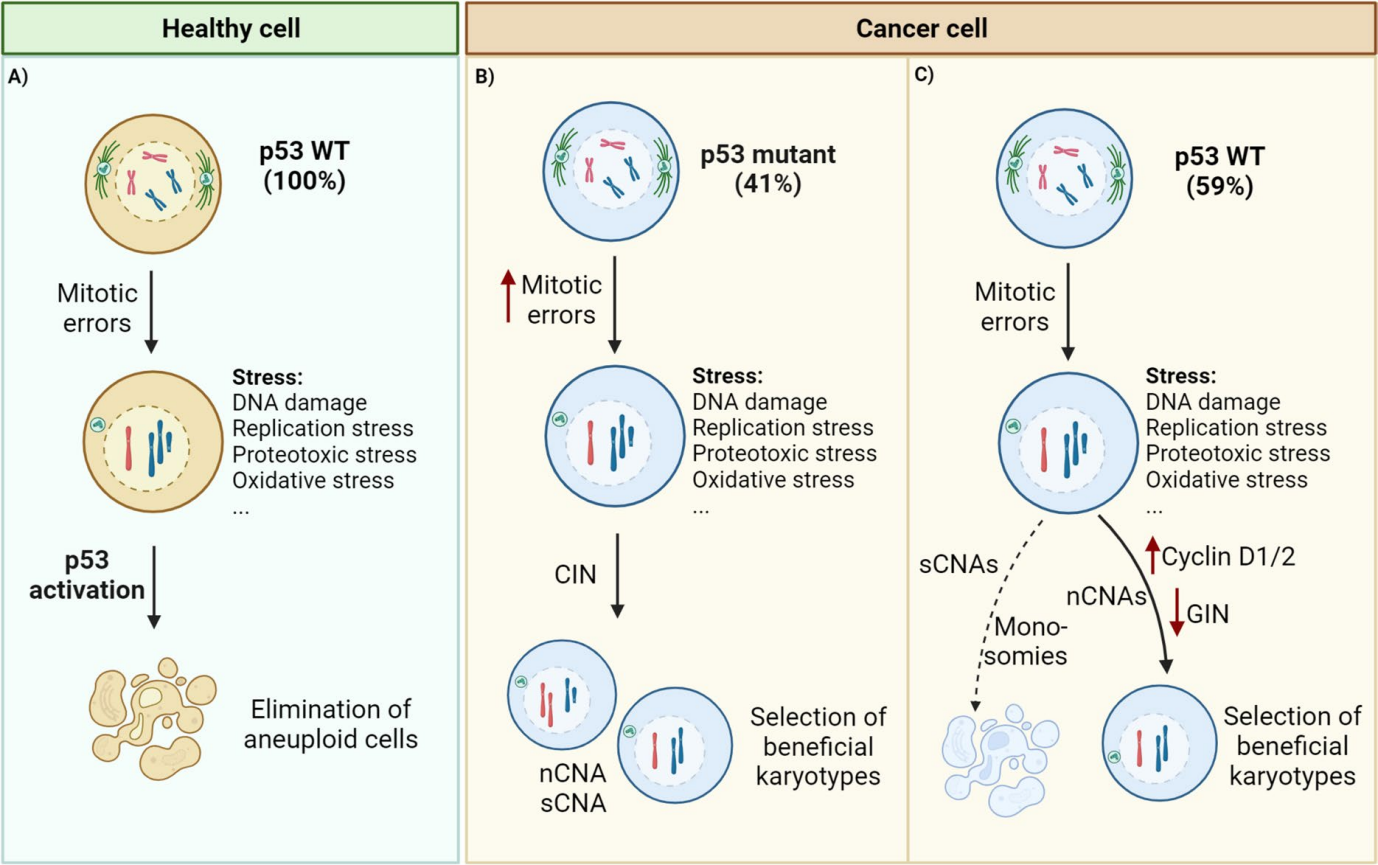
P53 as a gatekeeper for aneuploidy. In healthy cells (**A**), aneuploidy-induced stresses activate a p53 response, which prevents the propagation of aneuploid cells and causes their cell cycle arrest or death. In cancer cells, however, aneuploidy as well as p53 mutations are highly prevalent. In p53 mutant cancer cells (**B**) (~ 41% with *TP53* gene mutations) (Hoadley et al. 2014), aneuploid cells can proliferate with low/moderate levels of aneuploidy. Propagation of replication stress, for example, can induce DNA damage and consequently further promote CIN. This leads to increased heterogeneity, which enables selection of optimal karyotypes. Approximately 59% of tumors are WT for p53 (**C**). In these tumors, aneuploidy can still be propagated by either developing mechanisms to surpass p53 activation (e.g., cyclin D1/2 upregulation in WGD) or by upregulating mechanisms to prevent accumulation of genomic instability (GIN) (e.g., upregulate DNA damage response pathways). As a result, such cells are more likely to accumulate numerical CNAs (nCNAs), mostly gains, while cells with structural CNAs (sCNAs) and/or monosomies are probably eliminated or overtaken by cells with more beneficial karyotypes

P53 participates in a complex network of cellular responses to diverse stresses. As we discuss in this review, many of these stresses overlap with the ones induced by aneuploidy. It is clear that p53 activation is a recurrent outcome of aneuploidy but not an obligatory one as, for example, nCNAs can propagate in p53 proficient cells. Nevertheless, loss of p53 creates a more permissive context for proliferation of aneuploid cells when compared to p53 proficient counterparts (Salehi et al. 2021; Adell et al. 2023; Fujiwara et al. 2005). Therefore, there is still much to learn about how exactly aneuploidy triggers p53 and how p53 limits survival of aneuploid cells (Fig. 5). Some outstanding questions remain: first, does p53 get activated in response to aneuploidy in vivo? The fact that loss of p53 in vivo promotes survival of aneuploid cells suggests a role for p53, but direct evidence for p53 activation in response to aneuploidy in vivo is scarce. Given the absence of p53 response in mouse- and human-derived 3D organoid cultures, it will be important to examine direct p53 activation in aneuploid cells in 3D organoid cultures and validate it in in vivo models of CIN/aneuploidy. Second, it is currently unknown whether, and if so how, different types of mitotic errors (e.g., lagging chromosomes, chromatin bridges, supernumerary centrosomes, micronuclei) trigger p53 activation. A major distinction seems to be between errors that lead to sCNAs vs. nCNAs: whereas nCNAs are still tolerated to some extent in cells with functional p53, sCNAs often arise in p53-deficient cells. It will therefore be very informative to re-assess the correlation between *TP53* mutations and nCNAs. Third, it is still largely elusive whether p53 can work as a direct sensor for aneuploidy. Can p53, for example, be directly activated by a mis-segregating chromosome? Or is it perhaps more indirectly activated when a threshold is exceeded of the fraction of the genome that is altered (e.g., when more than 20% of the genome is gained or lost)? It could be of added value to use directed whole chromosome mis-segregation strategies such as KaryoCreate (Bosco et al. 2023) to explore whether p53 responds to a threshold of mis-segregation events (e.g., gene content, fraction of genome altered), in the absence of DNA damage. Lastly, it is also unresolved how different types of aneuploidy-induced stresses result in p53 activation. A direct link has been established between DNA damage responses and occurrence of sCNAs, but no other stresses were individually tested as mandatory intermediates for p53 activation in aneuploid cells. Understanding what mechanisms limit proliferation of aneuploid cells can open up therapeutic opportunities to target aneuploid tumor cells.

Most consequences of aneuploidy seem to impinge on the p53 pathway. However, p53 alterations are present at a pan-cancer frequency of approximately 41% while aneuploidy is present in ~ 90% of solid tumors, as obtained from TCGA database (Hoadley et al. 2014; Gerstung et al. 2020). This suggests that p53-proficient cells can propagate aneuploidy by either overcoming p53 activation or through p53-independent mechanisms (Fig. 5). For example, tetraploid human tumors (47% of which are wild-type for p53) (Crockford et al. 2017) can upregulate cyclin D1/2 which in turn sequesters p21, a downstream effector of p53, resulting in continued proliferation (Potapova et al. 2016; Crockford et al. 2017). Likewise, loss of BRG1, part of a chromatin remodeling complex, also seems to overcome p53 activation in aneuploid cells by upregulating cyclin D1 (Schiavoni et al. 2022). In another example, upregulation of HIF-1α can inhibit post-mitotic apoptosis and potentiate tolerance to aneuploidy in a p53-independent manner (Simoes-Sousa et al. 2018). Even if p53 itself is intact, alterations to components of the p53 network can also allow cells to overcome p53 pathway activation. For example, *CDKN2A* (encoding for p21) is a known cancer driver, frequently altered at early stages of tumor development (Donehower et al. 2019; Gerstung et al. 2020). Additionally, selection of sCNAs or nCNAs that affect genes involved in the p53 network can be another way by which aneuploid cells surpass p53 activation. In line with this, trisomy of chromosome 1q, recurrently present in cancer, leads to overexpression of MDM4 that in turn inhibits p53 function (Girish et al. 2023). Interestingly, gain of chromosome 1q was shown to be mutually exclusive with mutations in *TP53*. Likewise, deletion of *CDKN2A* and amplification of *MDM2* are mutually exclusive with *TP53* alterations in glioblastoma multiforme cancers (Donehower et al. 2019). Finally, since aneuploid cells can trigger further genomic instability (Sheltzer et al. 2011; Passerini et al. 2016), another way to adapt to aneuploidy might be by preventing accumulation of stresses (Clemente-Ruiz et al. 2016; Clarke et al. 2022). For example, aneuploid cells can improve DNA replication by activating DDK-mediated firing and mitotic DNA synthesis (MIDAS), in order to cope with replication stress and maintain mitotic fidelity (Garribba et al. 2023). It will be of great interest to examine in detail the general response to aneuploidy in p53 proficient cells and the pathways that such cells use to overcome aneuploidy’s detrimental effects. Given the prevalence of aneuploidy in cancer, such insights will likely reveal new targetable vulnerabilities of aneuploid cells.

## Data Availability

Data sharing is not applicable to this article as no datasets were generated or analyzed during the current study.

## Abbreviations

53BP1: P53-binding protein 1
aCGH: Array comparative genomic hybridization
ALT: Alternative lengthening of telomeres
AML: Acute myeloid leukemia
ATM: Ataxia-telangiectasia mutated
BCL9L: B-cell CLL/lymphoma 9 ligand
BE: Barrett’s esophagus
BRG1: Brahma-related gene-1
BUB1: Budding uninhibited by benzimidazoles 1
CDK2: Cyclin-dependent kinase 2
CENP-E: Centromere protein E
cGAS/STING: Cyclic GMP–AMP synthase/stimulator of interferon genes
CHK1/2: Checkpoint kinase 1/2
CIN: Chromosomal instability
CNA: Copy number alterations
DCB: Dihydrocytochalasin-B
DDK: DBF4-dependent kinase
FISH: Fluorescence in situ hybridization
GIN: Genomic instability
HIF-1α: Hypoxia inducible factor 1 subunit alpha
hMO: Human mammary organoids
IF: Immunofluorescence
KO: Knockout
LATS1/2: Large tumor suppressor 1/2
LOH: Loss of heterozygosity
mCO: Mouse colon organoids
MDH1: Nucleocytoplasmic malate dehydrogenase-1
MDM2: Murine double minute 2
MDM4: Murine double minute 4
MEFs: Mouse embryonic fibroblasts
M-FISH: Multiplex fluorescence in situ hybridization
MIDAS: Mitotic DNA synthesis
nCNA: Numerical copy number alterations
P53: Tumor protein P53
ROS: Reactive oxygen species
SAC: Spindle assembly checkpoint
sCNA: Structural copy number alterations
shRNA: Short hairpin RNA
siRNA: Small interfering RNA
TCGA: The Cancer Genome Atlas
TP53: Tumor protein 53 gene
TREX1: Three prime repair exonuclease 1
USP28: Ubiquitin specific peptidase 28
WGD: Whole-genome doubling
WT: Wild-type
